# Electroanalysis of Biochemistry and Material Chemistry

**DOI:** 10.3390/molecules30081687

**Published:** 2025-04-10

**Authors:** Guangjin Wang

**Affiliations:** School of Materials and Energy, Foshan University, Foshan 528000, China; wgj501@163.com

## 1. Introduction

Electrochemistry, which is an interdisciplinary science, has been applied in many fields [1]. Electrochemical technologies such as electrodeposition, electroreduction and electrospinning are a popular choice for the preparation of a wide range of materials, due to their advantages of mild reaction conditions, controllable processes, adjustable parameters and eco-friendliness [2,3]. Electroanalysis methods based on the detection of electrical signals, which offer the advantages of fast response and high sensitivity, have been applied to evaluate the electrochemical stability and electrocatalytic activity of various materials [4,5]. For the preparation and electroanalysis of high-performance materials, different kinds of electrochemical devices, such as fuel cells, supercapacitors and Li-ion batteries, have been developed [6,7,8]. In addition, through the combination of electrochemistry with other disciplines, such as optics and computer science, the fields of photoelectrochemistry and computational electrochemistry have emerged. Photoelectrochemical technologies have been leveraged to provide solutions to environmental pollution [9], and computational electrochemical methods have been employed to explore the mechanisms involved in electrochemical reactions and to design novel electrochemical materials [10]. This Special Issue of *Molecules*, “Electroanalysis of Biochemistry and Material Chemistry”, aims to collate the latest research in electrochemistry and its related fields. Herein, a brief overview of the papers published in this Special Issue is provided, summarizing their respective findings.

## 2. An Overview of the Published Articles

Wang et al. (contribution 1) fabricated biomimetic, three-dimensional, sponge-filled, nanofibrous nerve guidance conduits, preparing a polymeric (L-lactide-co-glycolide) membrane using electrospinning technology and synthesizing a collagen matrix using a freeze-drying method. The porosity, pore size and tensile strength of the polymeric membrane, as well as the crosslinking degree, porosity and pore structure of the collagen matrix, were studied, and the in vitro biocompatibility of the polymeric membrane and collagen matrix was evaluated. The results showed that the composite biomimetic nerve guidance conduits have broad application prospects for the repair of defects in long nerves. Liu et al. (contribution 2) synthesized gallium-doped hydroxyapatite nanoparticles using a chemical precipitation method, and discussed the effect of gallium ions on their crystal growth and morphology.

Zhao et al. (contribution 3) deposited an FeCrMoSi amorphous coating onto a 304 stainless steel substrate using an atmospheric plasma spraying process. The physicochemical properties and corrosion resistance of the amorphous coating were characterized. The results showed that boosting the surface roughness of the substrate improved the adhesion force between the amorphous coating and the substrate. The introduction of the Mo element significantly increased the hardness of the amorphous coating. The formation of an amorphous phase was conducive to decreasing the surface energy of the amorphous coating and enhancing its hydrophobicity. Because of these outstanding physicochemical properties, the coating exhibited excellent corrosion resistance and considerable interfacial contact resistance under the simulated operating conditions of a proton exchange membrane fuel cell. In addition, Zhao et al. (contribution 4) also reported that the commercial alloy Hastelloy χ displayed good mechanical properties, on account of its single γ-phase with a typical face-centered cubic structure. To explore the possibility of using Hastelloy χ as the bipolar plate in proton exchange membrane fuel cells, water contact angle measurement and electrochemical characterization techniques were employed to analyze its hydrophobicity and corrosion resistance. It was found that the water contact angle of Hastelloy χ was 79.5°, and that the charge transfer resistance of Hastelloy χ was 47.58 Ω cm^−2^. More importantly, the interfacial contact resistance of Hastelloy χ was 7.4 mΩ cm^−2^, which was almost one-twentieth that of 304 stainless steel (144.8 mΩ cm^−2^) under the same testing conditions.

Li et al. (contribution 5) prepared NiTi alloys using a suction casting method. The mechanical properties, corrosion resistance and interfacial contact resistance of the alloys were characterized. The hardness of the alloys was 289.7 HV, much higher than that of pure metals. Due to their higher open-circuit potential and lower corrosion current densities, the alloys exhibited higher corrosion resistance than that of pure Ni and Ti under the simulated operating conditions of a proton exchange membrane fuel cell. Moreover, the interfacial contact resistance of the alloys was 16.8 mΩ cm^−2^, which was attributed to the high concentration of metallic Ni in the alloys. These newly-fabricated alloys show promise as novel materials for the development of bipolar plates in proton exchange membrane fuel cells.

Zhao et al. (contribution 6) investigated the electrochemical performance of (La,Sr)(Co,Fe)O_3−δ_ cathodes under high carbon dioxide concentrations by using electrochemical impedance spectroscopy at different operating temperatures. They found that the introduction of carbon dioxide caused an obvious decrease in the electroconductivity of the cathodes, because carbon dioxide molecules were adsorbed and occupied the active sites on the surface of the cathodes, and thus limited the exchange rate of oxygen molecules. Fortunately, the blocking effect caused by the adsorbed carbon dioxide on the surface of the cathodes was reversible. For this reason, it was concluded that the cathodes exhibited excellent corrosion resistance under high carbon dioxide concentrations and different operating temperatures.

Fu et al. (contribution 7) summarized several modification methods to improve the electrocatalytic activity and electrochemical stability of perovskite oxides during oxygen reduction/evolution reactions under alkaline conditions, and proposed a combined method of experimental investigation and theoretical calculation to investigate the oxygen reduction/evolution mechanism of oxides and design high-performance oxides for oxygen reduction/evolution reactions.

Chen et al. (contribution 8) proposed a revised grand canonical potential kinetics method on the basis of the conventional density functional theory calculation model, deriving a calculation method which they then used to investigate the impact of surface charges on the kinetics and thermodynamics mechanism of the hydrogen evolution reaction on the surface of platinum. According to the calculation results, the optimal adsorption energy of hydrogen atoms on the surface of platinum is about −0.2 eV, which is attributed to the accumulation of electrons on the surface of platinum.

Zhong et al. (Contribution 9) assembled a flexible composite electrolyte, using Li_1.5_Al_0.5_Ge_1.5_(PO_4_)_3_ particles prepared using conventional solid-phase synthesis methods as filler, and investigated its Li^+^ conduction performance in all-solid-state batteries. The electrochemical analysis results showed that the flexible composite electrolyte exhibited high Li^+^ conduction and excellent electrochemical stability. The discharge capacity of the all-solid-state batteries was found to be 140 mA h g^−1^ at an overpotential of 0.15 V.

Chen et al. (contribution 10), using a chemical deposition method, deposited Ag-doped CdS nanoparticles onto S-doped graphitic carbon nitride to construct a Z-scheme heterostructure. The results showed that the synergistic effect of the doping of Ag atoms and the formation of the Z-scheme heterostructure was conductive to accelerating the transportation and separation of the photogenerated carriers, and thus to improving their photocatalytic performance for the degradation of Rhodamine B and methyl orange. Long et al. (contribution 11) used a combination of the sol–gel method and the spin coating process to prepare TiO_2_ films with different thicknesses. According to the results of photoelectrochemical measurements, the films with a thickness of 1.09 μm displayed the lowest photoelectrochemical reaction energy barrier and the fastest kinetics, due to these films exhibiting the highest short-circuit current density and open-circuit potential. In addition, the optimal photoelectrochemical parameters for the photoelectrocatalytic degradation of methyl orange on the surface of the TiO_2_ films were investigated. Under the optimized parameters, the degradation rate of methyl orange on the surface of the best film samples reached 88.6%, which was attributed to the interaction between reactive oxygen species and methyl orange.

Yang et al. (contribution 12) reviewed the use of photoelectrocatalytic water splitting for hydrogen production, covering a range of topics, from reaction mechanisms to semiconductor materials. They also proposed four effective methods to improve the yield of photoelectrocatalytic hydrogen production: morphological control, metallic/nonmetallic heteroatom doping, heterostructures and surface treatment.

## 3. Conclusions

This Special Issue highlights the latest research on the applications of electrochemical, photoelectrochemical and computational electrochemical technologies in the fields of biomimetic biology, material synthesis, computational materials and environmental chemistry. The studies included in this Special Issue employed electrochemical preparation methods to synthesize high-performance molecular polymers for use as biomedical materials. Several electroanalysis methods, such as linear potential scanning, cyclic voltammetry and electrochemical impedance spectroscopy, were applied to reveal the relationship between the corrosion resistance of alloys/oxides and their geometric structure and surface components, and to explore the performance of various oxides in electrocatalytic oxygen reduction/evolution. Electrochemical computational methods were used to uncover the mechanisms underlying the electrocatalytic activity of noble metals in hydrogen evolution reactions. Photoelectrochemical technologies were utilized to investigate the electrochemical performance of oxides/heterojunctions in organic dye degradation, and to analyze the mechanism involved in water splitting on semiconductor materials. The findings presented in this Special Issue provide a useful reference for researchers working in the fields of electrochemistry, photoelectrochemistry and computational electrochemistry.
